# Bi_2_Te_3_ Thin Films Deposited by the Combination of Bi and Te Plasmas in a PLD Process

**DOI:** 10.3390/mi14030590

**Published:** 2023-02-28

**Authors:** Laura A. Reyes-Verdugo, C. D. Gutiérrez-Lazos, J. Santos-Cruz, A. Chávez-Chávez, J. G. Quiñones-Galván

**Affiliations:** 1 Centro de Investigación en Ciencias Físico Matemáticas, Facultad de Ciencias Físico Matemáticas, Universidad Autónoma de Nuevo León, Av. Universidad s/n. Ciudad Universitaria, San Nicolás de los Garza 66455, Mexico; 2 Facultad de Química, Materiales-Energía, Universidad Autónoma de Querétaro, Queretaro 76010, Mexico; 3 Departamento de Física, Centro Universitario de Ciencias Exactas e Ingenierías, Universidad de Guadalajara, Boulevard Marcelino García Barragán 1421, Guadalajara 44430, Mexico

**Keywords:** thin films, pulsed laser deposition, plasma parameters, bismuth telluride

## Abstract

Bismuth telluride thin films were grown by pulsed laser deposition by implementing a novel method that combines both Te and Bi plasmas resulting from the laser ablation of individual Bi and Te targets. Furthermore, the mean kinetic ion energy and density of the plasmas, as estimated by TOF curves obtained from Langmuir probe measurements, were used as control parameters for the deposition process. The obtained thin films exhibit a metallic mirror-like appearance and present good adhesion to the substrate. Morphology of the thin films was observed by SEM, yielding smooth surfaces where particulates were also observed (splashing). Chemical composition analysis obtained by EDS showed that apparently the films have a Te-rich composition (ratio of Te/Bi of 3); however, Te excess arises from the splashing as revealed by the structural characterization (XRD and Raman spectroscopy). The XRD pattern indicated that depositions have the rhombohedral ($D_{3d}^{5}$ ($R\bar{3}m)$) structure of Bi_2_Te_3_. Likewise, Raman spectra exhibited the presence of signals that correspond to $E_{g}^{2}$, $A_{1u}^{2}$ and $A_{1g}^{2}$(LO) vibrational modes of the same rhombohedral phase of Bi_2_Te_3_. Additionally, oxidation states, analyzed by XPS, resulted in signals associated to Bi^3+^ and Te^2-^ that correspond to the Bi_2_Te_3_ compound. Finally, surface topology and thickness profiles were obtained from AFM measurements, confirming a combination of a smooth surface with particulates on top of it and a film thickness of 400 nm.

## 1. Introduction

Bismuth telluride (Bi_2_Te_3_) is a semiconductor material with a bandgap energy that varies in the range of 0.15 to 0.2 eV [1,2]. The crystal structure (rhombohedral), belonging to $D_{3d}^{5}$($R\bar{3}m)$ space group, has an atomic arrangement based on Te^(1)^–Bi–Te^(2)^–Bi–Te^(1)^ layers, where the superscripts denote different states of the Te anions. This means that each neutral plane of Bi_2_Te_3_ structure is built by five monatomic planes bonded by covalent bonds along the C axis [1,3]. The Te–Bi bonds are polar, while the bonds between the Te^(1)^–Te^(1)^ planes are weaker than those of the polar type. Consequently, the two weakly bonded big ions, the telluride ions, will be highly polarizable, property that makes Bi_2_Te_3_ a widely studied material for photocatalytic and thermoelectric applications [4,5]. Therefore, the synthesis process for Bi_2_Te_3_, will highly influence its structure, morphology, chemical state, and size; properties that are of important interest in the above-mentioned technological areas.

Concerning the Bi_2_Te_3_ synthesis methods, the material was prepared successfully using techniques based on chemical reaction in solution, such as electroless deposition [6], electrochemical deposition [7], solvothermal [1], and sol–gel [8], among others. Such techniques require additional treatments to obtain a uniform and adherent thin film, free of secondary phases formed by the employed chemical precursors. On the other hand, techniques based on physical vapor processes have also been successfully applied, among which we can mention the evaporation techniques such as pulsed laser deposition (PLD), lithography, spray pyrolysis, among others. The PLD technique, based on the laser ablation of solids, is a top-down method that takes advantage of the high energy of a pulsed laser to evaporate practically any type of materials, which allows its application in a great variety of fields. We can summarize the process of pulsed laser deposition of thin films in three stages, the assembly of ions, clusters and particles generated by the ablation process, nucleation, and growth.

Specifically, the laser ablation consists in the remotion of material from the surface of a solid by the application of short duration laser pulses of the order from 10^−13^ to 10^−8^ s, and power densities from 10^6^ to 10^14^ W/cm^2^ on the surface of the ablated target [9]. The removed material, also known as plasma plume, consists in a mixture of charged and neutral particles (electrons, ions, clusters, molecules, particulates) that will expand far from the surface and perpendicular to the ablated target. The ablation process can be carried out in vacuum, or in a gas or in a liquid environment, taking care that gas or liquid does not significantly attenuate energy or laser light intensity incident on the surface of the solid. This surrounding medium will determine the expansion and properties of the plasma. It is generally accepted that the resulting mean kinetic energies of the ions, within the interval of 1 to 300 eV, would allow the transport of material from the target to the substrate [10] to deposit a congruent thin film from compound targets. However, several considerations should take into account when preparing experiments regarding ablation of compound targets in order to diminish deviations in chemical composition of the targets and thin films. These considerations are highly dependent on the thermodynamic and optical absorption properties of the target. In some cases, loss of high volatile elements could be expected during the plasma expansion towards the target, making the congruent deposition a difficult task. To overcome this issue, plasma diagnosis has become a powerful tool that allows for shedding light on the mean kinetic energy and density of the arriving species on the substrate. Both parameters play an important role in the final composition, and thus properties of the growing films [11,12,13].

The electronic layer of the active plasma close to the surface substrate has a positive effect on the adhesion coefficient. The use of inert gas allows a reduction in the re-evaporation of volatile components of the films. An attractive aspect of this technique is its capacity for the tank of vapors at high pressures of reactive gases. This method has been used successfully to obtain thin Bi_2_Te_3_ films at temperatures around 300 °C [14].

To our knowledge, all works in literature reporting pulsed laser deposition of Bi_2_Te_3_ use a single compound target for the ablation process, which ends up in dealing with strategies to overcome deviations from the stoichiometry of the target in the final composition of the films along with elevated substrate temperatures or post-annealing treatments [14,15,16,17,18,19].

In the present work, bismuth telluride thin films were grown using PLD, combining both Te and Bi plasmas. Combination of plasmas to deposit compound or doped thin films controlling the chemical composition by means of plasma diagnosis tools has been previously reported in several works by our group [12,13,20,21,22,23,24]. The morphological, structural, chemical composition, and topological properties were studied for a fixed combination of Te/Bi ion density ratio.

## 2. Materials and Methods

**Figure 1:**
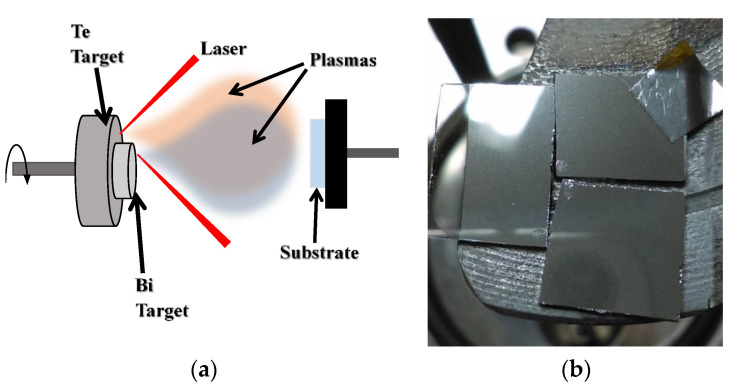
(**a**) PLD system configuration. (**b**) Deposited films.

Bismuth telluride thin films were grown using the pulsed laser deposition method. The deposition process was achieved by the combination of plasmas resulting from the simultaneous ablation of high purity Te and Bi targets. Figure 1a shows a schematic diagram of the PLD system. The Te target was obtained from the compression of high purity powders into a 1-inch pellet, while the Bi target was obtained from the melting of high purity Bi pellets in an inert atmosphere to avoid oxidation in a ½-inch pellet. Afterwards, the Bi pellet was attached coaxially to the Te target using double-sided carbon tape.

For the laser ablation process a Nd:YAG laser emitting at 1064 nm with an output energy of 600 mJ per pulse, 6 ns duration pulses and repetition rate of 10 Hz, was used. The beam was divided into two equal beams using a 50–50 beam splitter. Each laser beam was focused on one of the targets while they were rotating at 15 rpm to avoid cumulative ablation in the same spot leading eventually to a drilling process. The target to substrate distance for Bi was 4.7 cm, while for Te it was 5 cm. The deposition chamber was evacuated to a base pressure of 3 × 10^−6^ Torr, which was also the working pressure. For the deposition process, glass slides were used as substrates keeping a deposition time of 20 min. Three substrates were used in a single experiment in order to evaluate uniformity of the films. The configuration of the substrates is shown in Figure 1b. On the right side of the substrate holder, two substrates with area of 1 cm^2^ were placed, and at the left side a substrate of 1 × 1.5 cm^2^. Thickness of the films was measured at the corners of the substrates, and it was found that an approximate area of (1.5 × 1.5) cm^2^ at the center of the array can be considered as uniform in thickness with a value of 400 nm. Regarding morphology, no substantial changes were observed. Additionally, due to the configuration of the targets, as it could be expected, the zones at the extremes sides of the array differed in chemical composition as one extreme was Bi rich while its counterpart was Te rich. In spite of the latter, at the center of the array, a uniform chemical composition was obtained. Regarding structural properties, the individual substrates were characterized, finding no substantial differences in the uniform observed zones.

**Figure 2:**
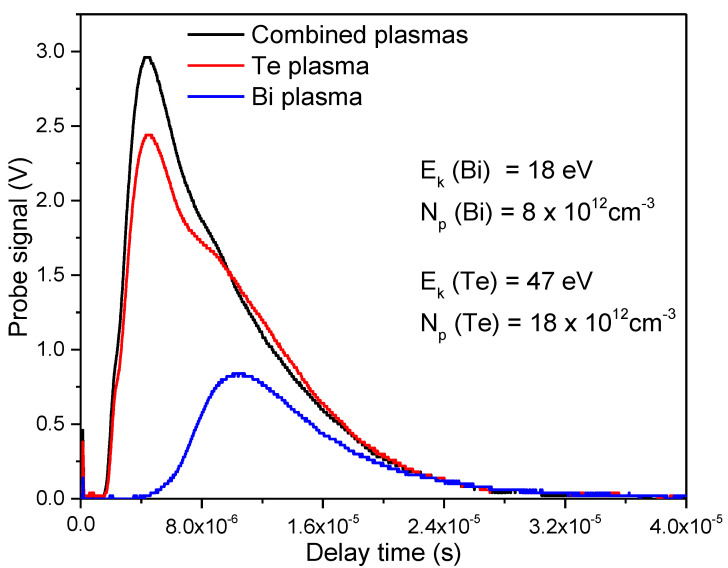
TOF curves for Bi and Te plasmas.

Prior the deposition of the films, the individual Te and Bi plasmas were diagnosed by means of time of flight (TOF) measurements using a planar electrostatic Langmuir probe with 6 mm of diameter biased to −48 V, from which mean kinetic energy and ion density were obtained. For TOF measurements, the voltage drop across a 20 Ohm resistance was detected using a Tektronix 500 MHz digital oscilloscope. The calculation of mean kinetic energy of the ions uses the following relationship [25]:

$$〈E_{k}〉=\frac{mL^{2}}{2}\frac{\int_{0}^{T}t^{-2}I\left(t\right)dt}{\int_{0}^{T}I\left(t\right)dt}$$

where m is the mass of the ion, L is the target to probe distance, and $I\left(t\right)$ is the probe current as a function of time. For the plasma ion density calculation, the following equation was used [20,26]:

$$N_{p}=\frac{I_{max}}{evA},$$

where *I_max_* stands for the maximum value of the current (saturation current), e is the electron charge, v is the plasma flow velocity and A is the area of the probe. The obtained TOF curves with mean kinetic energy and density of Bi and Te ions is shown in Figure 2.

From Figure 2, it can be noticed that the plasma density ratio for Te and Bi was 2.25, which is higher than the Te/Bi ratio in Bi_2_Te_3_. This higher ratio in the plasmas was chosen in order to compensate possible Te losses during the experiment aiming to obtain stoichiometric films. The use of individual Te and Bi targets to produce separated plasmas, that can be combined while expanding towards the substrate instead of a stoichiometric Bi_2_Te_3_ target, is preferred in this work, owing that required specific compositions can be more easily controlled by means of the plasma diagnosis tool. The fact of having separate individual beams impinging each target, allows manipulation of incident energy on the individual targets, which make that desired ion densities could be chosen searching for avoiding deviation from stoichiometry. In contrast, the use of stoichiometric targets would need several different experiments in order to find adequate conditions to obtain nearly stoichiometric films. Because Bi and Te have different vapor pressures, ablating with a single beam would necessarily induce Te losses that need to be compensated, or instead, avoided by the use of inert gases at higher pressures, compromising the deposition rate and structural properties of the films. Although it is accepted that laser ablation intrinsically produces congruent deposition of thin films from compound targets, this should be taken with care due it not being straightforward [25].

The surface morphology of the films was observed by scanning electron microscopy (SEM), using a JEOL JSM-6010LA microscope. Measurements were carried out at a 20 kV acceleration voltage. Chemical composition was studied by energy dispersive X-ray spectroscopy using an EDS detector attached to the SEM microscope. Structural characterization was carried out by X-ray diffraction using an empyrean system from PANALYTICAL (Malvern, UK) and Raman spectroscopy with a Thermo Scientific DXR2 system (Thermo Fisher Scientific Inc., Waltham, MA, USA) equipped with a 633 nm laser as excitation source. The oxidation states were studied using an X-ray photoelectron spectroscopy in a Flex-mod SPECS system (SPECS GmbH, Berlin, Germany) equipped with a PHOIBOS 100 detector. Surface topology and films thickness were measured by atomic force microscope using an Agilent Technologies system (Agilent Technologies, Inc., Santa Clara, CA, USA) model 5420 in contact mode.

## 3. Results and Discussion

### 3.1. Scanning Electron Microscopy

**Figure 3:**
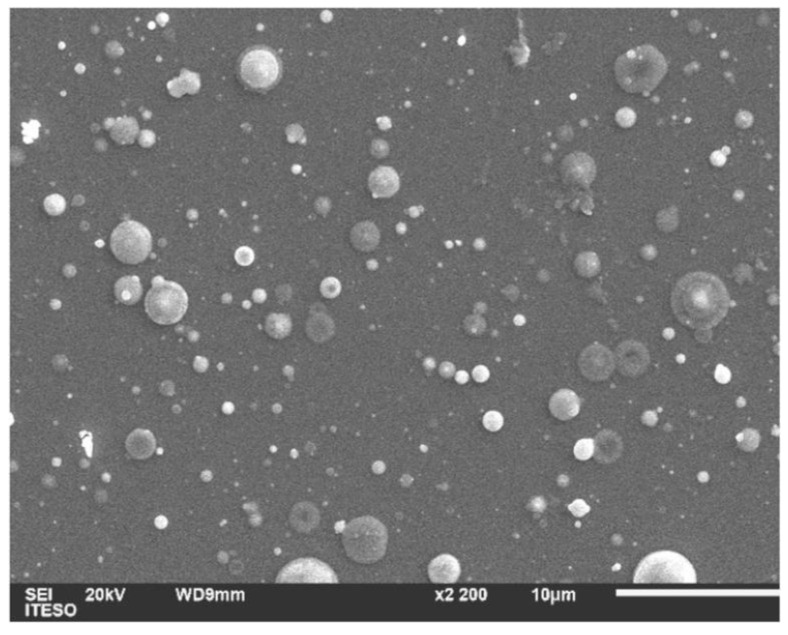
Scanning electron micrographs of Bi_2_Te_3_ film deposited by PLD technique.

In Figure 3, a typical film surface micrograph from PLD of Bi and Te targets is shown. According to Dauscher et al. [15], different types of particulates may be observed when depositing Bi_2_Te_3_ with PLD, originating from target surface roughening as it has already been reported [27,28] and identified as defects produced due the laser irradiation (thermal and mechanical shocks). This is the well-known splashing phenomenon, one of the greatest disadvantages of the PLD technique. As discussed below, the EDS study confirms the presence of elemental Bi and Te at the surface of the films resulting from splashing of individual targets. Nevertheless, the surface of the films that is free from particulates appears to be dense and smooth.

### 3.2. EDS Analysis

**Figure 4:**
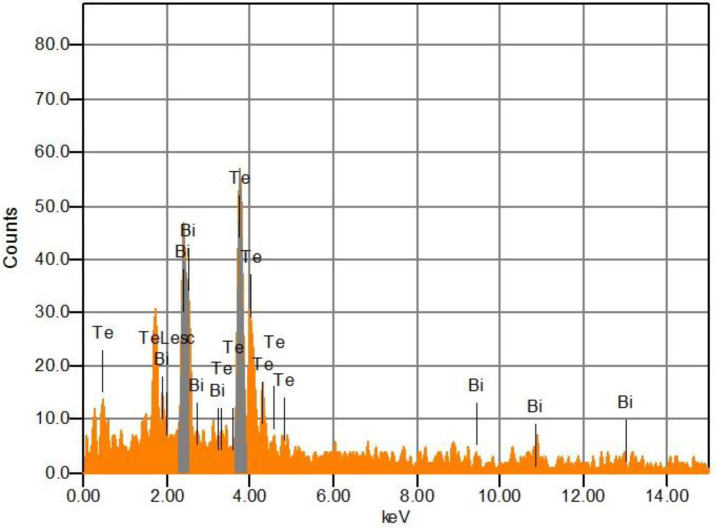
EDS spectrum of Bi_2_Te_3_ film deposited by PLD technique.

Figure 4 shows a representative EDS spectrum for the Bi_2_Te_3_ film. Spectra for the films were recorded in different zones of the samples. Punctual measurements on individual particulates showed that they are composed either by a high percentage of Bi or Te. For the study presented here measurements of observed surfaces (10 × 10 µm^2^) were carried out at several regions of the samples; however, we present results corresponding to the central zone of the array shown in Figure 1b. Solely signals corresponding to Bi and Te are present, the atomic percentage for Bi and Te elements resulted in 25 and 75%, respectively, which correspond to a Te/Bi ratio of 3, which is considerably higher than the 2.25 ratio used in the plasma densities of Te and Bi. Considering a stoichiometric Bi_2_Te_3_ compound, atomic Te/Bi ratio should be 1.5; however, the apparent discrepancy between EDS composition results and stoichiometric Te/Bi ratio arises from the splashing mentioned above. Particulates of elemental Te and Bi contribute to the chemical composition determined by EDS. We see in the structural analysis and XPS results that the composition of film beneath the particulates is near to the desired stoichiometric compound.

### 3.3. XRD Analysis

**Figure 5:**
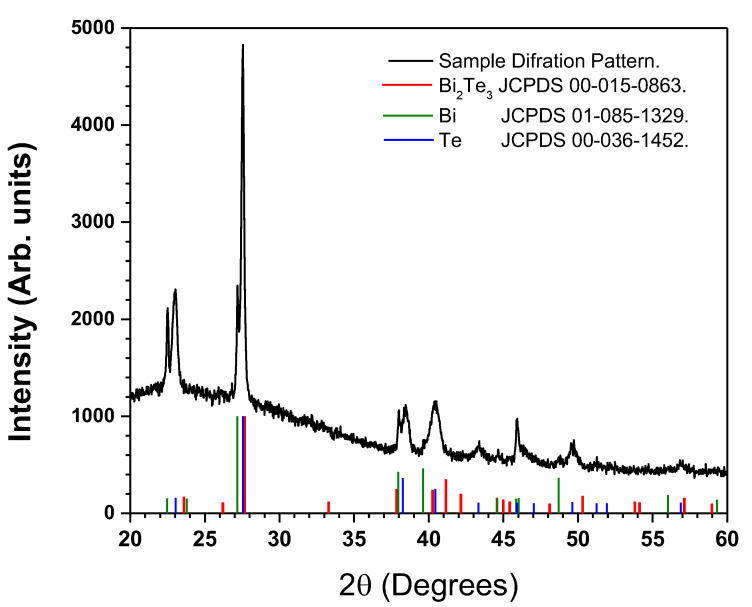
XRD Analysis of synthesized Bi_2_Te_3_ film.

In Figure 5, the diffraction pattern obtained from a polycrystalline thin film deposited by the simultaneous ablation of Bi and Te targets is exhibited. The colored lines represent the reference standards for Bi_2_Te_3_ (red), Bi (green), and Te (blue) structures, according to the JCPDS-ICDD files 00-015-0863, 01-085-1329, and 00-036-1452, respectively. Depending on the film deposition technique, the intensity of the peaks may vary [27]; however, the preferred growth plane of all Bi_2_Te_3_ is shown at the (0 1 5) plane, around to 27.54°. A characteristic peak of films prepared by PLD is shown at the (0 0 6) plane, confirming that it is feasible to obtain at room temperature a film with a higher degree of crystallinity, in comparison with other deposition methods [29,30]. Note that the appearance of signals corresponding to elemental Bi and Te confirms the presence of splashing at the surface of the films, which is in accordance with SEM and EDS discussions. Crystallite size for Bi_2_Te_3_ was calculated using the Scherrer equation as ~55 nm, which is in good agreement with the Takashiri report [31], in which Bi_2_Te_2.7_S_0.3_ was synthesized by means of the flash evaporation method. According to their discussion, nanocrystalline thin films based on bismuth telluride can potentially have improved thermoelectric responses due to quantum effects arising from size reduction. Therefore, obtaining small grain size (less than 200 nm) in bismuth telluride thin films is important for a possible application on thermoelectric devices with improved figure of merit [31,32,33]. Nevertheless, the present work is mainly focused on the synthesis method and the resulting structural, morphological, and chemical composition of the thin films.

### 3.4. Raman Analysis

**Figure 6:**
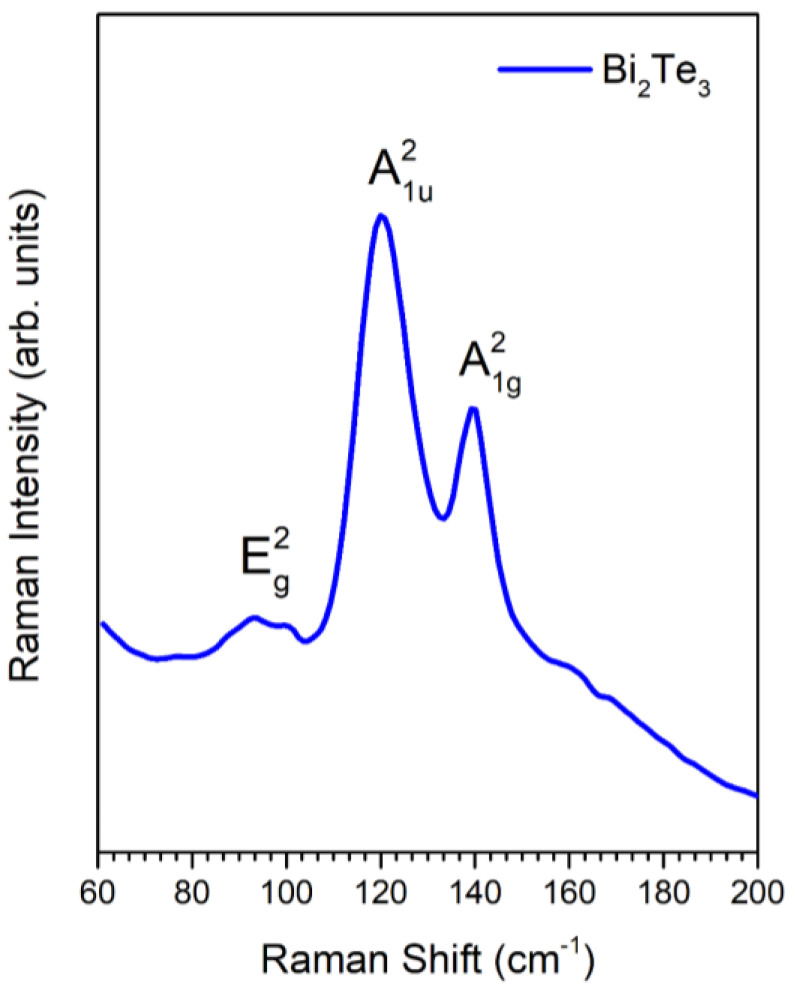
Raman spectrum of synthesized Bi_2_Te_3_ film.

A typical Raman spectrum of a synthesized Bi_2_Te_3_ thin film is shown in Figure 6. The presence of signals centered at 94, 120, and 139 cm^−1^, assigned to the $E_{g}^{2}$, $A_{1u}^{2}$, and $A_{1g}^{2}$ (LO) vibrational modes, respectively, corresponding to the rhombohedral-structured Bi_2_Te_3_, which are consistent with reports in the literature [31,32,33,34] and the XRD analysis.

As can be seen in Figure 6, mode $E_{g}^{2}$ is twofold degenerate in $E_{g}^{2}$, which means that the atoms vibrate in the basal plane. On the other hand, notice that the $A_{1u}^{2}$ mode appears with the highest intensity in Figure 6. The appearance of $A_{1u}^{2}$ mode has been reported when crystal symmetry breaking occurs in nanoplates or nanosheets [1,2]; thus, it could be possible that bismuth telluride thin films of the present work are composed of thin structures stacked together to form crystallites with a 55 nm size.

### 3.5. XPS Analysis

**Figure 7:**
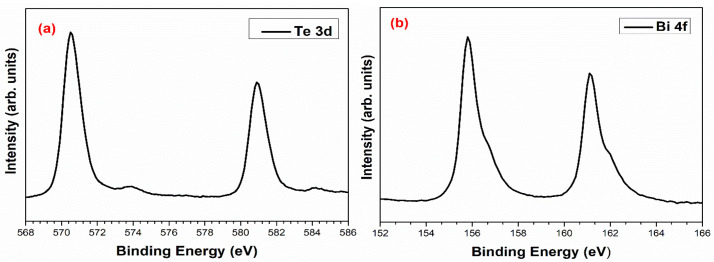
XPS spectra for (**a**) Te 3d and, (**b**) Bi 4f of synthesized Bi_2_Te_3_ thin film.

Figure 7 shows the XPS spectra of the Bi_2_Te_3_. The Te spectrum, Figure 6a, shows two sets of peaks at 570.82 and 580.94 eV, which correspond to Te^−2^ 3d_5/2_ and Te^−2^ 3d_3/2_. It also has small peaks at 573.88 and 584.11 eV due to surface oxidation. A previous study reported that nanostructured materials are more easily oxidized [35,36,37]. Figure 6b shows the high-resolution scan of Bi 4f doublet peaks centered at 155.78 eV and 161.10 eV, corresponding to Bi^+3^ 4f_7/2_ and Bi^+3^ 4f_5/2_, respectively, confirming the formation of the Bi_2_Te_3_ phase [37,38,39,40].

### 3.6. AFM Analysis

**Figure 8:**
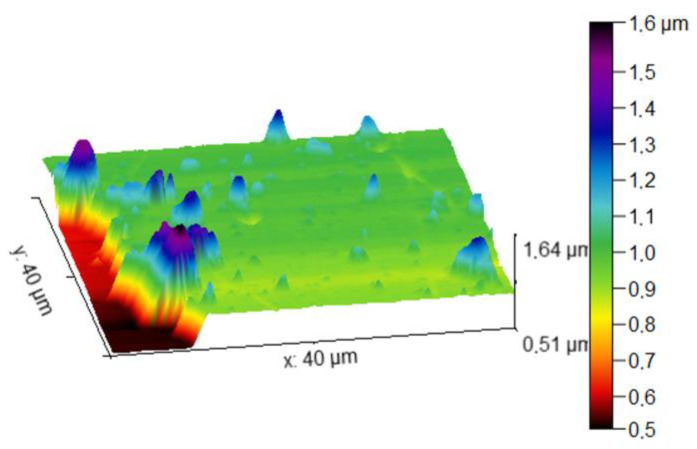
AFM micrograph for Bi_2_Te_3_ thin film.

The thickness and surface topography of the films were determined using AFM measurements. Figure 8 shows an atomic force micrograph of the Bi_2_Te_3_ sample. Profile measurements at zone protected during the deposition to prevent material growth show that the thickness of the film is approximately 400 nm, which means that a deposition rate of 20 nm/min was achieved, which is important for thermoelectric applications.

The surface topology of the film shows that the areas that are free from splashing are dense and smooth, which is consistent with the SEM results. The presence of particulates can be seen through all the surfaces.

## 4. Conclusions

A simple way to elaborate bismuth telluride films by PLD with an innovative method using elemental Bi and Te Targets has been presented. Since there is a big difference between the bismuth and tellurium vapor pressures, obtaining satisfactory stoichiometry could be a challenge, but with convenient experimental conditions, it is feasible to achieve crystalline stoichiometric thin films even on substrates at room temperature. The XRD, EDS, and XPS results proved the crystallinity and chemical composition of the films, respectively. Raman studies confirmed the presence of Bi_2_Te_3_ characteristic vibrational modes. It can be concluded that the combination of laser-produced plasmas from the laser ablation of individual elemental targets demonstrated to be a powerful technique for the deposition of complex systems such as Bi_2_Te_3_, for which applications are highly sensitive to elemental composition. Furthermore, the use of Langmuir planar probes for plasma diagnosis allows stoichiometry control as well as experimental reproducibility.

## Data Availability

The data presented in this study are available on request from the corresponding author.
